# Rational Molecular Design of Aniline‐Based Donor‐Acceptor Conducting Polymers Enhancing Ionic Molecular Interaction for High‐Performance Wearable Bioelectronics

**DOI:** 10.1002/adhm.202501929

**Published:** 2025-07-04

**Authors:** Junning Qian, Ruya Shi, Li Zhang, Wing Cheung Mak

**Affiliations:** ^1^ Department of Biomedical Engineering The Chinese University of Hong Kong Shatin, New Territories Hong Kong 999077 China; ^2^ Shun Hing Institute of Advanced Engineering The Chinese University of Hong Kong Shatin, New Territories Hong Kong 999077 China

**Keywords:** conducting polymers, donor‐acceptor interaction, molecular engineering, polyaniline, wearable bioelectronics

## Abstract

Organic conducting polymers (CPs) are crucial for wearable bioelectronic devices, including biosensors, offering enhanced sensitivity and adaptability for personalized health monitoring. However, the classical CPs molecular structure limits their development of high‐performance wearable biosensors. Among all, pH monitoring related to H^+^ ions is important for managing metabolic disorders, monitoring wound healing and various skin diseases. Here, a novel concept is demonstrated for fabrication of high‐performance donor‐acceptor (D‐A) aniline‐based CPs with enhanced sensitivity and stability for wearable biosensors. Density functional theory (DFT) calculations are implemented, using aniline as the acceptor and ortho‐substituted aniline derivatives as donors. This approach enables precise molecular design of the CPs molecular structure through different conjugated units. Guided by the DFT calculations, donor‐donor (D‐D) and D‐A/CPs, specifically poly(aniline‐co‐o‐fluoroaniline) (P(ANI‐co‐FANI)) and poly(aniline‐co‐o‐methoxyaniline) (P(ANI‐co‐MOANI)), are synthesized and examined. The high‐performance D‐A/P(ANI‐co‐MOANI) exhibits a significant 1.4‐ and 3.7‐fold increase in sensitivity and 3.6‐ and 9.0‐fold enhancement in stability for pH sensing compared to PANI and D‐D/P(ANI‐co‐FANI). Real‐time sweat pH monitoring is further demonstrated with the advanced D‐A/P(ANI‐co‐MOANI)‐based wearable pH biosensor during various activities. These findings provide critical insights into designing and synthesizing advanced functional CPs for innovative high‐performance organic bioelectronic devices, including sensing, drug delivery, and energy‐related applications.

## Graphical abstract

Organic conducting polymers are crucial for advanced wearable bioelectronic devices. A novel aniline‐based donor‐acceptor conducting polymer is developed via a rational molecular design approach, exhibiting enhanced ionic molecular interactions for highly sensitive and stable wearable pH sensors. This work offers a new molecular design strategy for next‐generation functional conductive polymers, enabling high‐performance wearable bioelectronics.

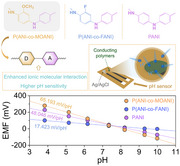

## 1 Introduction

With the rising demand for personal health management, especially facing the global challenge of the ageing population with increasing demands on healthcare, the development of wearable bioelectronic devices such as biosensors for decentralized health monitoring has garnered significant attention in recent years.^[^ ^1^, ^2^, ^3^, ^4^ ^]^ As they can provide a promising alternative for early disease diagnosis and real‐time health monitoring due to their lightweight design, flexibility, and comfort.

As an advanced functional material, conducting polymers (CPs) have become attractive for developing high‐performance wearable biosensors owing to their advantageous properties, such as organic nature, soft, flexible and biocompatible, which are ideal for on‐body wearable bioelectronic devices.^[^ ^5^, ^6^, ^7^ ^]^ Beyond that, CPs are essential in the wearable biosensors designed for sensing ionic molecules (Na^+^, K^+^, Ca^2+^, Mg^2+^, H^+^, etc.), attributed to their unique tunable electronic/ionic properties, the sensing performance of which can be quantified through the mechanism of ionic interactions, such as doping and dedoping ions within the CPs.^[^ ^8^, ^9^ ^]^ In addition, ion doping has also been proven to enhance the long‐term stability of CPs‐based devices.^[^ ^10^ ^]^ Through ionic doping strategy, the doping degree of CPs could be enhanced, forming a more stable conjugated structure that reduces the impact of external environmental factors.^[^ ^11^ ^]^ Furthermore, ion doping could enhance the conductivity of conducting polymers by increasing the carrier concentration.^[^ ^12^ ^]^ Sweat is an attractive biological fluid that contains a rich array of ionic molecules such as Na^+^, K^+^, H^+^, as well as glucose, lactate, urea, small protein molecules for non‐invasive health monitoring.^[^ ^13^, ^14^, ^15^ ^]^ Among different ionic molecules, H^+^ ions concentration related to pH‐level is particularly important. As fluctuations in pH can reflect the body's metabolic status and serve as indicators of certain metabolic diseases, such as cystic fibrosis, alkalosis, and skin diseases caused by Candida albicans infection.^[^ ^16^, ^17^, ^18^ ^]^

To date, a variety of wearable pH sensors have been developed using different analytical technologies, ranging from electrochemical and colorimetric, to fluorescence methods.^[^ ^19^ ^]^ However, most conventional pH sensors are neither flexible nor durable, making them not ideal for real‐time monitoring of pH. Flexible and wearable pH sensors are particularly valuable for modern healthcare and medical applications, enabling real‐time, continuous, and personalized health monitoring, which is significant for disease prevention and improving quality of life.^[^ ^20^ ^]^ As the core part of wearable pH sensors, the materials used in the sensing module directly affect the sensitivity and stability of these sensors. Among the developed pH sensing materials, polyaniline (PANI) is almost an exclusive choice in the reported literature. It manifests excellent electrical activity, low cytotoxicity, and low skin irritation.^[^ ^21^ ^]^ During the doping process of proton acid in PANI, hydrogen ions (H^+^) move onto the molecular chain of PANI, causing protonation of nitrogen atom on the C = N group. This process significantly enhances the conductivity of PANI, enabling it to transition reversibly from an insulating to a conductive state.^[^ ^22^, ^23^, ^24^ ^]^ Compared to metal‐based pH sensors, flexible pH sensor devices composed of PANI are lighter and more convenient for wearable biomedical devices. These physicochemical characteristics make PANI well‐suited for flexible and wearable pH sensors. Though it has been developed and applied to flexible pH sensors for over two decades. In recent significant achievements in bioelectronics manufacturing, researchers remain enthusiastic about using PANI in pH sensor modules for flexible bioelectronics,^[^ ^20^, ^25^, ^26^ ^]^ indicating the broad market prospects of PANI in biomedical applications. Undoubtedly, PANI is an excellent material for fabricating flexible pH sensors. However, the limited pH sensing performance of pure PANI cannot meet the growing market demand for future wearable pH sensors. To address this, significant efforts have been made to construct PANI‐based heterocomposites to overcome these limitations, including PANI/Au,^[^ ^27^ ^]^ F‐Ti_3_C_2_T_x_/PANI,^[^ ^28^ ^]^ PANI/PPy,^[^ ^29^ ^]^ MoS_2_‐PANI,^[^ ^30^ ^]^ PANI‐PEDOT:PSS,^[^ ^31^ ^]^ PANI/CuO,^[^ ^32^ ^]^ etc. However, most developed wearable pH sensors are based on the well‐known structure of PANI, involving loading or coupling PANI as the core unit without altering its molecular structure, which is the key underlying the pH sensing mechanism. Consequently, these constructions do not deviate from the nature of PANI, restricting the diversified development of PANI‐based pH sensors. To address these limitations, we investigate the reversible H^+^ doping/dedoping mechanism of PANI at the molecular level, design and construct a diverse PANI‐based molecular framework from a molecular engineering perspective with enhanced pH sensing performance.

The challenge in developing efficient, wearable PANI‐based pH sensors through molecular engineering lies in the chemical characteristics of the PANI backbone, which interacts minimally with cationic ions like H^+^.^[^ ^6^ ^]^ Besides, the designed molecular framework must enhance the doping capacity for H^+^ without disrupting the pH sensing mechanism of PANI. The features of conducting polymers offer an effective physicochemical strategy to keep the molecular backbone of PANI and enhance its pH sensing performance. Compared to PANI, a rational molecular design of aniline‐based conducting polymers can not only maintain the molecular skeleton of PANI but significantly improve the π–π^*^ stacking degree of benzene rings, thereby enhancing the electron transfer efficiency over molecular chains.^[^ ^33^, ^34^ ^]^ This requires, without altering the backbone of the PANI molecule, the rational arrangement for constructing aniline‐based conducting polymers would be to select aniline and its derivatives as corresponding monomers. Furthermore, in the molecular design of conducting polymers, constructing a donor‐acceptor (D‐A) typed structure can effectively optimize the energy levels of the highest occupied molecular orbital (HOMO) and lowest unoccupied molecular orbital (LUMO) of conducting polymers molecules, changing the polarity and electron distribution.^[^ ^35^ ^]^ By optimizing electron density and electrostatic potential on the conducting polymers molecular surface through resonance and inductive effects, the electronic transfer efficiency of the conducting polymers is promoted, thereby enhancing the reactivity of the conducting polymers.^[^ ^36^, ^37^ ^]^ Therefore, constructing a reasonable aniline‐based D‐A conducting polymers to customize the physicochemical properties of PANI is a promising approach to advancing the PANI structure and applying it to flexible pH sensors.

In this work, engineered aniline‐based conducting polymers of D‐A typed poly(aniline‐co‐*o*‐methoxyaniline) and D‐D typed poly(aniline‐co‐*o*‐fluoroaniline), simplified as P(ANI‐co‐MOANI) and P(ANI‐co‐FANI)), have been built by computer‐assisted molecular design through density functional theory (DFT) calculations method. It is found that D‐A typed P(ANI‐co‐MOANI) bearing methoxy group, efficiently regulates the electron density and electrostatic potential on the benzene ring, thereby significantly increasing the electron transfer rate in the molecular chain and promoting the adsorption ability of the C = N group toward cations. Herein, we have developed a new flexible pH‐sensitive aniline‐based D‐A conducting polymer, P(ANI‐co‐MOANI) for manufacturing a wearable pH sensor. The physicochemical properties and electrochemical properties of the P(ANI‐co‐MOANI) were systematically characterized. The synthesized P(ANI‐co‐MOANI) could be used for quantitative pH analysis, delivering excellent sensitivity and stability than PANI. Moreover, we further developed an integrated custom‐built three‐dimensional (3D) P(ANI‐co‐MOANI)‐based flexible and wearable pH sensor, and successfully achieved real‐time pH monitoring of sweat, demonstrating the broad prospects for biomedical applications. This is the first time using molecular design strategy to optimize the molecular surface electrostatic potential to regulate the electronic structure of the aniline‐based D‐A conducting polymers for wearable and high‐performance pH sensor through the strategy by modulating the H^+^ doping and dedoping ability.

## 2 Results and Discussion

### 2.1 Molecular Design of Aniline‐Based D‐A Conducting Polymers

**Figure 1:**
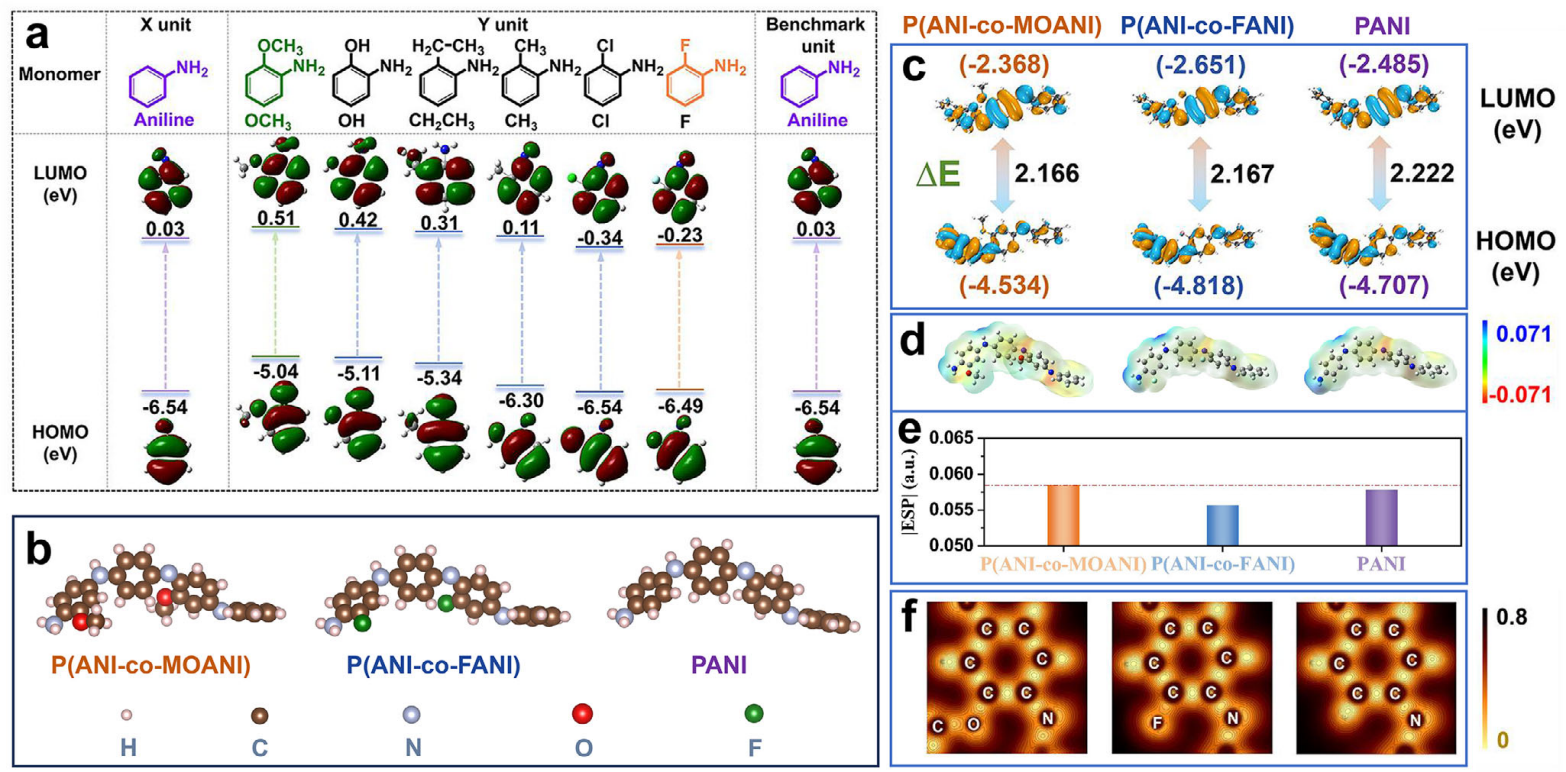
(a) LUMO and HOMO diagram of aniline and ortho‐substituted aniline derivatives for building conducting polymers; (b) Molecular structures with simplified unit of P(ANI‐co‐MOANI) (left), P(ANI‐co‐FANI) (middle) and PANI (right); (c) LUMO and HOMO diagrams of P(ANI‐co‐MOANI), P(ANI‐co‐FANI) and PANI; (d) ESP maps and (e) the lowest negative charge values and (f) LOL plots of benzene ring and C = N group in P(ANI‐co‐MOANI) (left), P(ANI‐co‐FANI) (middle) and PANI (right).

Ortho‐substituted aniline derivatives, having a molecular structure like aniline, are employed to construct aniline‐based conducting polymers (Figure S1, Supporting Information). Aniline is chosen as the acceptor module (X unit), and ortho‐substituted aniline derivatives are selected as the donor modules (Y unit) (**Figure** **1**a). Six different combinations (X+Y) are thereafter examined to build the possible D‐A conducting polymers. Theoretically, constructing aniline‐based D‐A conducting polymers requires that the donor and acceptor units have aligned frontier orbitals. The LUMO and HOMO of the donor module form a staggered structure with the HOMO and LUMO of the acceptor module, ensuring the HOMO and LUMO in D‐A conducting polymer are contributed by the donor and acceptor modules, accordingly.^[^ ^38^ ^]^ Another D‐D conducting polymer, mainly contributed by a single donor module, is constructed for the comparison. This D‐D conducting polymer consists of two donor units with sandwiched orbital energy alignment. Comparably, the D‐A conducting polymer is more effective in promoting electron transfer within the molecular chain, making it a suitable molecule for enhancing the pH sensing performance. To efficiently design D‐A conducting polymers, we analyze the LUMO and HOMO of different modules (Figure 1a). The results illustrate that the LUMO of aniline is higher than that of the modules with ‐OCH_3_, ‐OH, ‐CH_2_CH_3_, and ‐CH_3_ groups, while its HOMO is lower than those of them, forming a well‐staggered frontier orbitals structure required for the molecular design of D‐A conducting polymers. Among them, the OCH_3_‐functionalized module with the highest LUMO and lowest HOMO is selected as a representative for further analysis. We perform DFT calculations to evaluate the molecular orbital overlap analysis between donor and acceptor units (Figure S2, Supporting Information), its unique structure facilitates significant orbital overlap effects between donor and acceptor units. Moreover, aniline has higher LUMO and lower HOMO than those of F‐functionalized modules, allowing them to form D‐D conducting polymers. As for the Cl‐functionalized module, though its LUMO is higher than that of aniline, the HOMO of it is equal to that of aniline. Therefore, engineered molecules of D‐A conducting polymers P(ANI‐co‐MOANI), D‐D conducting polymers P(ANI‐co‐FANI), and benchmark (PANI) are then constructed and subjected to the next calculations (Figure 1b). 

Bandgap and HOMO energy level are two vital aspects to evaluate the structural characteristics and possible pH sensing ability of the conducting polymers. As shown in Figure 1c, both P(ANI‐co‐MOANI) and P(ANI‐co‐FANI) have lower theoretical bandgaps than PANI. This means the improved π–π^*^ stacking degree and stronger interaction in the conducting polymers molecules, demonstrating their promoted conjugated degree.^[^ ^39^, ^40^ ^]^ The enhancement is more obvious in the D‐A conducting polymers P(ANI‐co‐MOANI) due to its smaller theoretical bandgap. Importantly, P(ANI‐co‐MOANI) has a higher HOMO compared to P(ANI‐co‐FANI) and PANI, with the order being P(ANI‐co‐MOANI) (−4.534 eV) > PANI (−4.707 eV) > P(ANI‐co‐FANI) (−4.818 eV). The HOMO level is generally used to describe the molecular propensity to lose electrons.^[^ ^41^ ^]^ Evidently, the P(ANI‐co‐MOANI) molecule is more prone to electron loss and has lower reduction potential, also confirmed by experimental CV results (Figure S3, Supporting Information). When functionalized as a pH sensing material, P(ANI‐co‐MOANI) exhibits higher electron transport efficiency, attributed to its enhanced partial electron delocalization effect, predominantly via the thermally activated hopping mechanism (Figure S4, Supporting Information).^[^ ^42^, ^43^ ^]^ This facilitates easier H^+^ adsorption on the surface of the P(ANI‐co‐MOANI) molecule through electrostatic interactions, making it a promising pH sensing material compared to PANI.^[^ ^44^ ^]^ Conversely, P(ANI‐co‐FANI), with the lowest HOMO, may perform poorly as a pH sensing material compared to PANI due to its weaker electron transfer efficiency resulting from stronger electron localization, which hinders H^+^ interaction with the molecular surface.

To visually analyze the distribution of surface charges on different molecules, electrostatic potential (ESP) calculations are used (Figure 1d). Cool colors (green to blue) imply regions predominantly distributed with positive charges, while warm colors (yellow to red) represent areas where negative charges are concentrated.^[^ ^45^ ^]^ Among the different molecules, the ESP values around the nitrogen atoms of the C = N bond appear in yellow, affirming these regions are rich in negative charges and more likely to interact with H^+^.^[^ ^46^ ^]^ This area serves as the chemical basis for their function as pH‐sensing materials. From the color intensity, it is evident that P(ANI‐co‐FANI) has the lightest yellow color, supporting the weakest interaction with H^+^ among the three molecules. However, the yellow color intensities of P(ANI‐co‐MOANI) and PANI are relatively similar, making it difficult to distinguish their differences by color intensity alone. To further identify this difference, we quantitatively calculate their lowest ESP values, including that of P(ANI‐co‐FANI). As shown in Figure 1e, the absolute ESP values near the C = N bond follow the order: P(ANI‐co‐MOANI) (0.0585) > PANI (0.0578) > P(ANI‐co‐FANI) (0.0557), demonstrating that P(ANI‐co‐MOANI), with its enhanced interaction with H^+^, is more suitable as a pH sensing material than PANI and P(ANI‐co‐FANI). 

Although we have analyzed their potential characteristic in pH sensing, the reasons why the same benzene ring and C = N group structures result in significantly different negative charges remain unclear. Therefore, we implement the localized orbital locator (LOL) analysis (Figure 1f) to better understand the differences from the view of electronic structures.^[^ ^44^, ^47^ ^]^ The brown regions represent larger LOL values, while the yellow regions represent smaller LOL values. There are lone pairs of electrons around the N atom, as well as in the outer layer of the O atom in P(ANI‐co‐MOANI) and the F atom in P(ANI‐co‐FANI). N atoms in the C = N group with lone pair electrons are the binding sites for H^+^ binding. However, although both O and F atoms contain lone pair electrons, they demonstrate opposite effects in P(ANI‐co‐MOANI) and P(ANI‐co‐FANI). The electrons around the O atom mainly flow into the benzene ring (an interaction region appears between the C‐C and C‐O bonds), making the electron cloud closer to the benzene ring. In contrast, the F atom absorbs electrons from the benzene ring (an isolated region can be seen between the C‐C and C‐F bonds), making the electron cloud closer to the F atom. The simulation results unwire and elucidate the mechanism by which the D‐A effect influences the cationic H^+^ combination, providing new scientific insight into the molecular engineering design and application of aniline‐based D‐A conducting polymers in pH sensing.

### 2.2 Physicochemical Properties of Engineered Conducting Polymers

**Figure 2:**
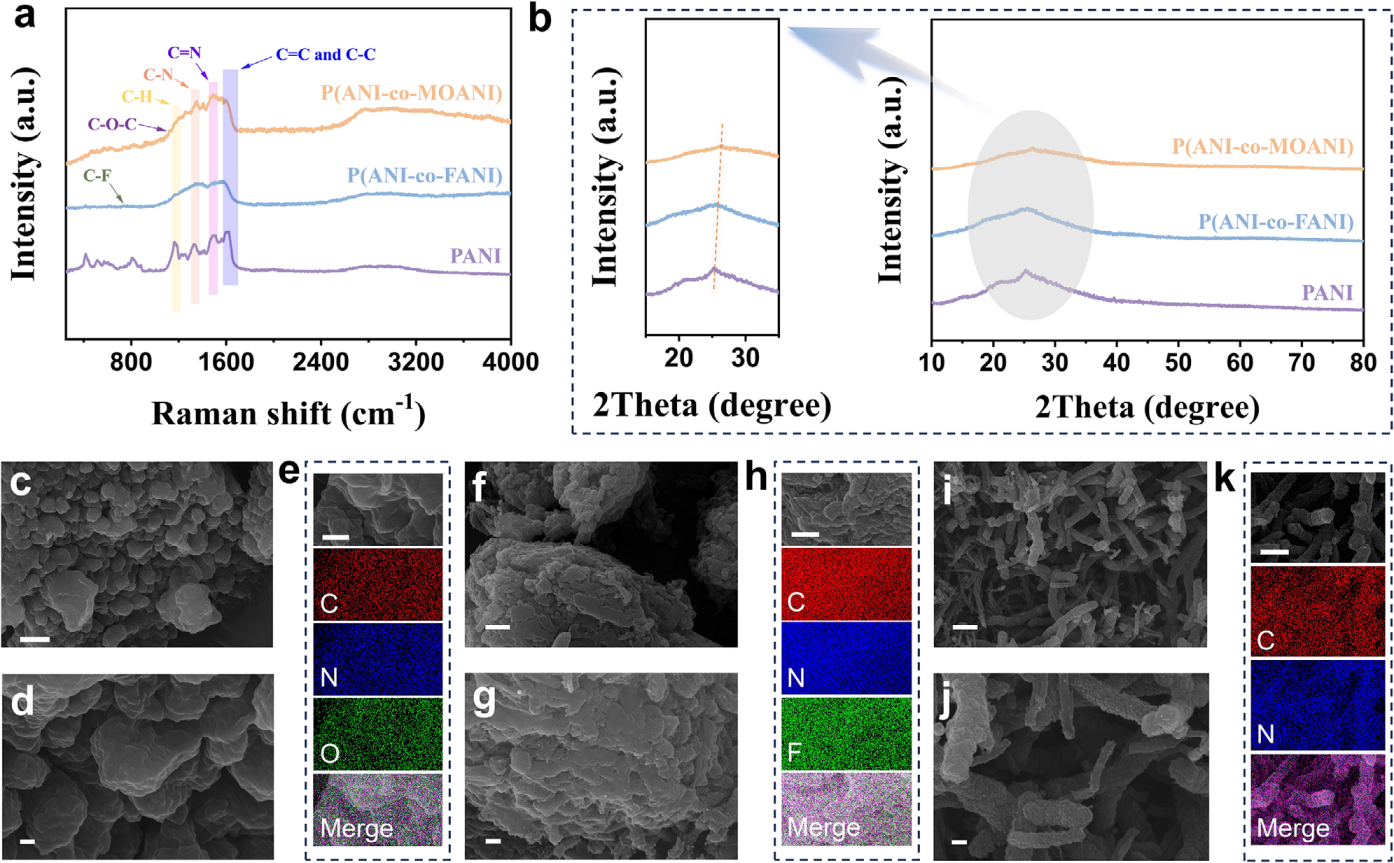
(a) Raman spectra and (b) XRD patterns of P(ANI‐co‐MOANI), P(ANI‐co‐FANI) and PANI; SEM images of (c) (d) P(ANI‐co‐MOANI), (f,g) P(ANI‐co‐FANI) and (i,j) PANI with the scalebar of 1 µm (top) and 200 nm (bottom), respectively; EDS‐Mapping results with the scalebar of 100 nm for (e) P(ANI‐co‐MOANI), including (Top: SEM image, Bottom: Corresponding elements distribution of C (carbon), N (nitrogen), O (oxygen) and Merge (the combined image of SEM and different elements distribution)); (h) P(ANI‐co‐FANI), including (Top: SEM image, Bottom: Corresponding elements distribution of C (carbon), N (nitrogen), F (fluorine) and Merge (the combined image of SEM and different elements distribution)); and (k) PANI, including (Top: SEM image, Bottom: Corresponding elements distribution of C (carbon), N (nitrogen) and Merge (the combined image of SEM and different elements distribution)).

Considering the theoretical calculation results, two representative conducting polymers, P(ANI‐co‐MOANI) and P(ANI‐co‐FANI), have been synthesized using the electropolymerization method. Additionally, PANI, employed as a benchmark, was prepared using the same method. The physicochemical characteristics of them are studied by Raman spectra and XRD patterns, respectively. Raman spectra (**Figure** **2**a) indicate the presence of various chemical bonds in the backbone and pendant groups of these aniline‐based copolymers, such as C‐H, C‐C, C‐N, and C = C bonds.^[^ ^48^, ^49^ ^]^ Notably, after undergoing extensive washing steps, the characteristic C‐O‐C and C‐F bonds in the Raman spectra, originating from the pendant groups of P(ANI‐co‐MOANI) and P(ANI‐co‐FANI), have been observed, respectively. Importantly, the characteristic C = N bond, which exists solely within the aniline‐based polymers backbone while absent in the individual monomers, are observed, suggesting the formation of aniline‐based CPs.^[^ ^50^ ^]^ EDS‐Mapping results show that these elements of C, N, O, and C, N, F and C, N are evenly distributed in P(ANI‐co‐MOANI) (Figure 2e), P(ANI‐co‐FANI) (Figure 2h), and PANI (Figure 2k), respectively. The broad XRD diffraction peak near 25° suggests the obtained conducting polymers and PANI are composed of amorphous phases (Figure 2b), consistent with the previous report.^[^ ^51^ ^]^ P(ANI‐co‐MOANI) denotes a red‐shift in the XRD diffraction peak compared to PANI and P(ANI‐co‐MOANI). This is primarily attributed to enhanced partial electron delocalization effects induced by D‐A interactions, resulting in changes in lattice parameters.^[^ ^52^, ^53^ ^]^ In addition, the increased electron transfer on the benzene ring, resulting from the D‐A effect, greatly promotes the interaction among molecular chains in P(ANI‐co‐MOANI), further enhancing overall structural orderliness.

Combining the morphological features of P(ANI‐co‐MOANI) (Figure 2c,d), P(ANI‐co‐FANI) (Figure 2f,g) and PANI (Figure 2i,j), the enhanced structural orderliness of P(ANI‐co‐MOANI) and P(ANI‐co‐FANI) is further confirmed. Unlike the random distribution of rod‐shaped PANI, P(ANI‐co‐MOANI) and P(ANI‐co‐FANI) consist of stacked nanoparticles, demonstrating their organized and compacted structures. The surface of P(ANI‐co‐MOANI) is smooth with uniformly distributed nanoparticles, while P(ANI‐co‐FANI) has a rough surface with irregular particles distribution. This demonstrates the P(ANI‐co‐MOANI) with D‐A effect facilitates the π–π^*^ stacking among molecular backbone chains and consequently leads to the formation of uniform nanostructures. In general, D‐A and D‐D conducting polymers are not simple mixtures of different polymers, and such significant differences in morphology have also been observed in previous reports.^[^ ^54^, ^55^ ^]^

### 2.3 pH Sensing Performance of Engineered Conducting Polymers

**Figure 3:**
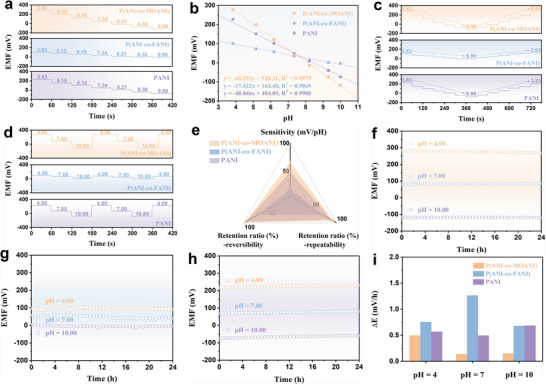
(a) OCP versus pH curves and (b) calibration plots of P(ANI‐co‐MOANI), P(ANI‐co‐FANI) and PANI (pH: 3.83‐9.99); OCP versus pH curves of P(ANI‐co‐MOANI), P(ANI‐co‐FANI) and PANI ranging in (c) 3.83‐9.99‐3.83 and (d) 4‐7‐10‐4‐7‐10‐4; (e) Radar plots of pH sensing performance of P(ANI‐co‐MOANI), P(ANI‐co‐FANI) and PANI; OCP versus time curves of (f) P(ANI‐co‐MOANI), (g) P(ANI‐co‐FANI) and (h) PANI (pH = 4, 7, 10); (i) Variation rates of OCP values during stability tests for P(ANI‐co‐MOANI), P(ANI‐co‐FANI) and PANI.

Due to the protonation effect of the C = N group, aniline‐based polymers can be doped with H^+^. To specify their differences in pH sensing, the OCP between the pH sensing materials modified WE and RE is measured across different pH levels. **Figure** **3**a,b displays that different pH sensing materials achieve high linearity (R^2^ > 0.98) over a wide pH range (4–10). The pH sensitivities of P(ANI‐co‐MOANI), P(ANI‐co‐FANI), and PANI are 65.193, 17.423, and 48.046 mV pH^−1^, respectively. This revealed that P(ANI‐co‐MOANI) is more sensitive to the change of pH conditions. Additionally, it also shows a fast response (<1 s) in pH sensing (Figure S5, Supporting Information). Figure 3c,d illustrate two models for pH variation, which are used to verify the pH reversibility and repeatability of different sensing materials. For the pH reversibility result, in the forward (pH varies from 4–10) and reverse (pH varies from 10–4) measurement, P(ANI‐co‐MOANI) maintains the initial signal of 98.7%. For the pH repeatability result, P(ANI‐co‐MOANI) retains the initial signal of 93.2% during multiple OCP measurements (pH varies from 4–10 for three times). However, P(ANI‐co‐FANI) and PANI exhibit relatively poor reversibility and repeatability, which only maintain 71.6%, 72.8%, and 96.6%, 89.5%, of their initial signals, respectively. To observe the performance differences of three pH sensing materials more intuitively, the radar plots including different pH sensing indicators of sensitivity, reversibility and repeatability are made for the comparison. It is evident the region surrounded by three sensing indicators of sensitivity, reversibility and repeatability for P(ANI‐co‐MOANI) is larger than those of P(ANI‐co‐FANI) and PANI in the triangular pattern (Figure 3e), further denoting the D‐A effect reinforced P(ANI‐co‐MOANI) delivers enhanced pH sensing performance in multiple aspects, while the P(ANI‐co‐FANI) with D‐D effect has the opposite influence and is not well‐performance in the pH sensing. Subsequently, stability tests over 24 h at different pH levels (pH = 4, 7, 10) are carried out (Figure 3f–i). The P(ANI‐co‐MOANI) exhibits a smaller OCP variation in 24 h with the drifting rates of 0.5 mV h^−1^ (pH = 4), 0.14 mV h^−1^ (pH = 7) and 0.15 mV h^−1^ (pH = 10), while the drifting rates of P(ANI‐co‐FANI) and PANI are 0.75, 1.26, 0.68 mV h^−1^ and 0.57, 0.50, 0.69 mV h^−1^ in pH = 4, 7, 10, appearing the promoted durability in pH sensing by D‐A effect reinforced P(ANI‐co‐MOANI). Notably, this advanced P(ANI‐co‐MOANI) material exhibits excellent sensitivity (65.193 mV pH^−1^), wide pH sensing range (4–10), fast response time (< 1s) and good stability (OCP drift: 3.36 mV/24 h) compared to other pH sensing composites (Table S1, Supporting Information). This demonstrates the successful design of the engineered D‐A conducting polymers in enhancing pH sensing performance and highlights its potential for future practical applications.

### 2.4 The Underlying Mechanism of pH Sensing in the Advanced D‐A Conducting Polymer of P(ANI‐co‐MOANI)

**Figure 4:**
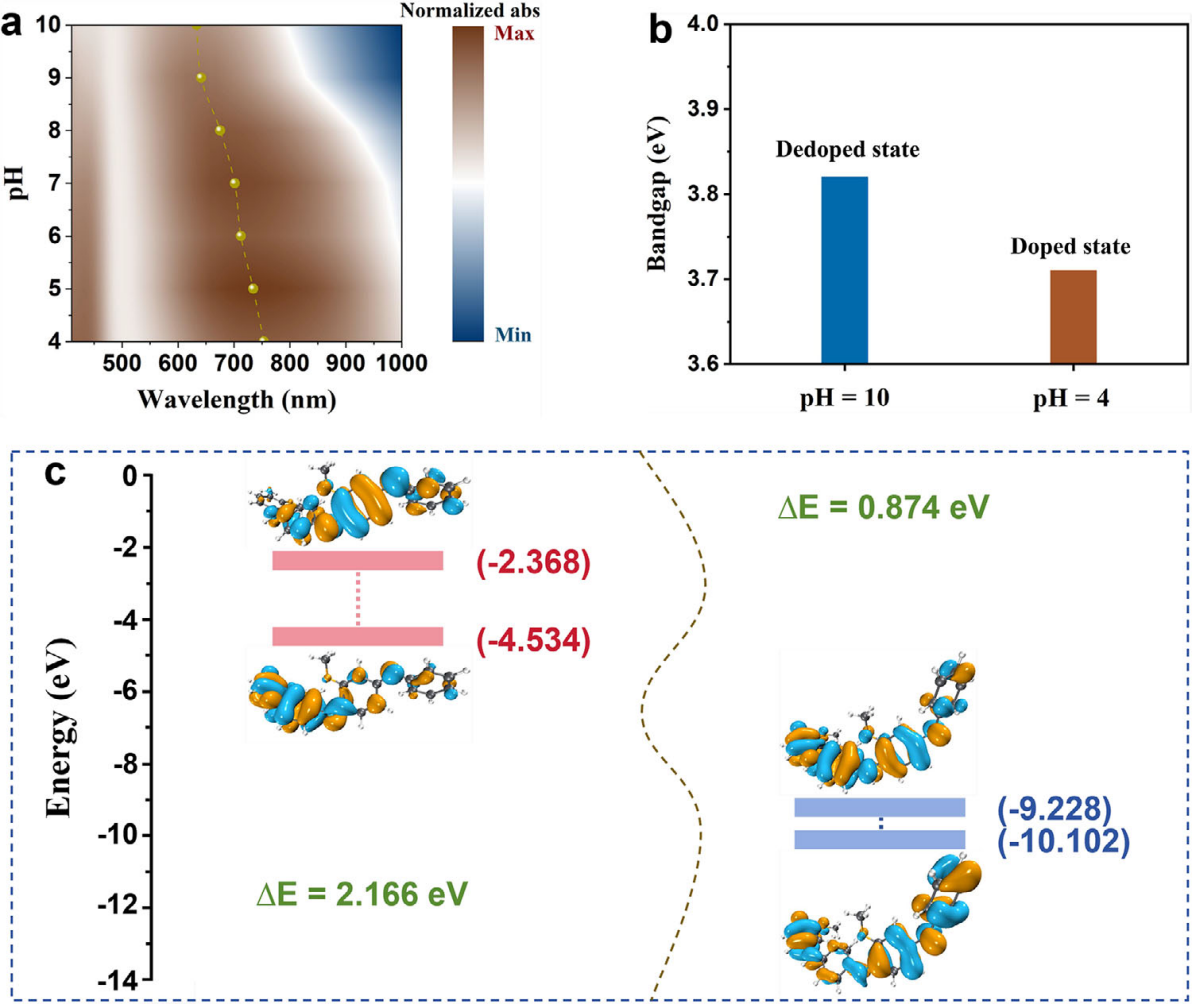
(a) UV–vis spectra of P(ANI‐co‐MOANI) between 10 and 4; (b) Bandgap values of P(ANI‐co‐MOANI) at pH 10 and 4; (c) Calculated bandgaps, frontier orbitals of P(ANI‐co‐MOANI) and its interaction with H^+^ ions.

It is evident that P(ANI‐co‐MOANI) exhibits outstanding pH sensing ability. Although the theoretical calculation has provided insights into the structural origin of P(ANI‐co‐MOANI) in pH sensing process, it remains experimentally unclear how H^+^ ions doping and dedoping affect P(ANI‐co‐MOANI) structure in acidic and alkaline conditions. Therefore, we further investigate the chemical reaction mechanism of P(ANI‐co‐MOANI) during the pH sensing process through varying pH values from 10 to 4. **Figure** **4**a gives the UV–vis spectra of P(ANI‐co‐MOANI) at pH levels ranging from 10 to 4. It exhibits two sets of peaks within 400–1000 nm: A shoulder peak at about 440 nm and a broad peak from 500 to 1000 nm. These peaks correspond to the polaron band transition and the quinone structure in the P(ANI‐co‐MOANI) backbone, respectively.^[^ ^56^, ^57^ ^]^ As the pH value reduces from 10 to 4, the peak intensity (440 nm) gradually increases, while the broad peak (500–1000 nm) undergoes a significant red shift. This reveals the amount of combined H^+^ ions on the C = N group increases with decreasing pH, causing the delocalization of polarons and enhancement in surface charge mobility and intrinsic conductivity of P(ANI‐co‐MOANI).^[^ ^58^ ^]^ This exactly explains that P(ANI‐co‐MOANI) exhibits a lower OCP signal in an alkaline environment compared to an acidic environment and also confirmed by surface resistances of P(ANI‐co‐MOANI) before and after the interaction with H^+^ ions (Figure S6, Supporting Information). Additionally, the broad peak (500–1000 nm) is getting wider in the protonation process, attributed to the increased doping degree and enhanced molecular stability.^[^ ^59^ ^]^ The sensing performance of CPs is closely related to their bandgap across various pH conditions.^[^ ^60^ ^]^ The band structure of P(ANI‐co‐MOANI) possibly changes with varying pH. The change in pH alters the H^+^ ions‐doping degree of CPs, which could be enhanced in acidic conditions (e.g., pH = 4), resulting in a decrease in the bandgap. This occurs because the H^+^ ions‐doping degree process introduces additional charge carriers into the molecules, which facilitates easier electron transitions from the valence band to the conduction band, thereby enhancing the material's conductivity.^[^ ^61^ ^]^ In contrast, the H^+^ ions‐doping degree of CPs could be significantly reduced in alkaline conditions (e.g., pH = 10), resulting in an increase in its bandgap. This makes electron transitions more difficult, consequently weakening the material's conductivity. Therefore, it is crucial to analyze the bandgap of CPs during the pH sensing process. Based on the theoretical results, different experimental bandgaps of P(ANI‐co‐MOANI), derived from Tauc plots (Figure S7, Supporting Information), at pH levels of 10 and 4 (Figure 4b) are compared. It supports that the reduced pH causes the decrease in the experimental bandgap of P(ANI‐co‐MOANI). DFT calculation results also confirm this observation (Figure 4c), for P(ANI‐co‐MOANI), after it combines with H^+^ ions, the distribution region of electrons in P(ANI‐co‐MOANI) is getting wider, leading to the enhancement of doping degree in P(ANI‐co‐MOANI).

### 2.5 Advanced P(ANI‐co‐MOANI)‐Based Wearable pH Sensor in Real‐Time pH Monitoring of Sweat

**Scheme 1:**
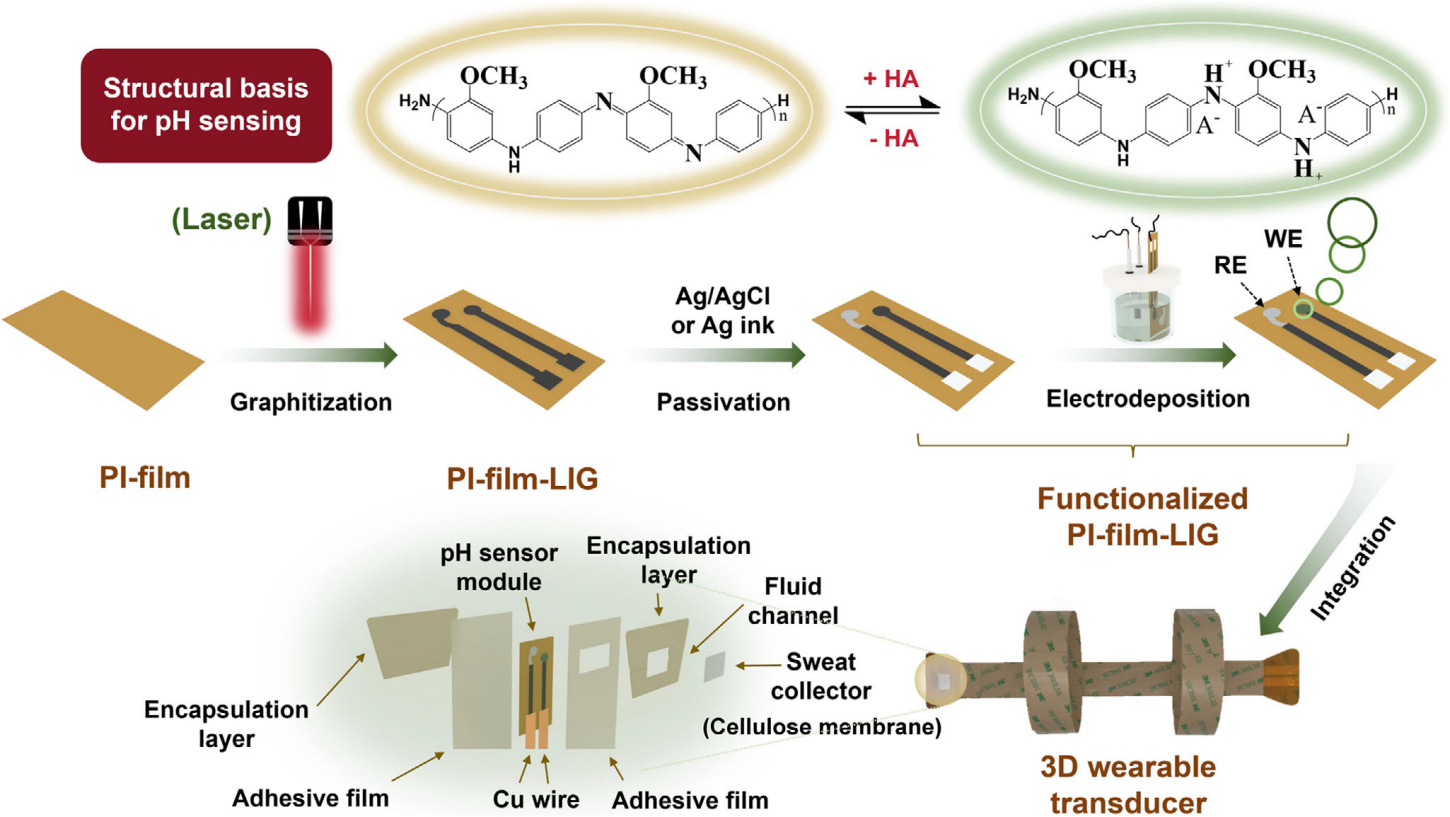
Schematic diagram of manufacturing flexible and wearable transducer using P(ANI‐co‐MOANI).

To further expand the practical application of P(ANI‐co‐MOANI) in real‐time pH monitoring of sweat, a P(ANI‐co‐MOANI)‐based flexible and wearable sensor has been designed and fabricated. **Scheme** **1** describes the entire manufacturing process, including the fabrication of WE, RE, fluid channel, and wearable components. Detailed descriptions can be found in the experimental section.

**Figure 5:**
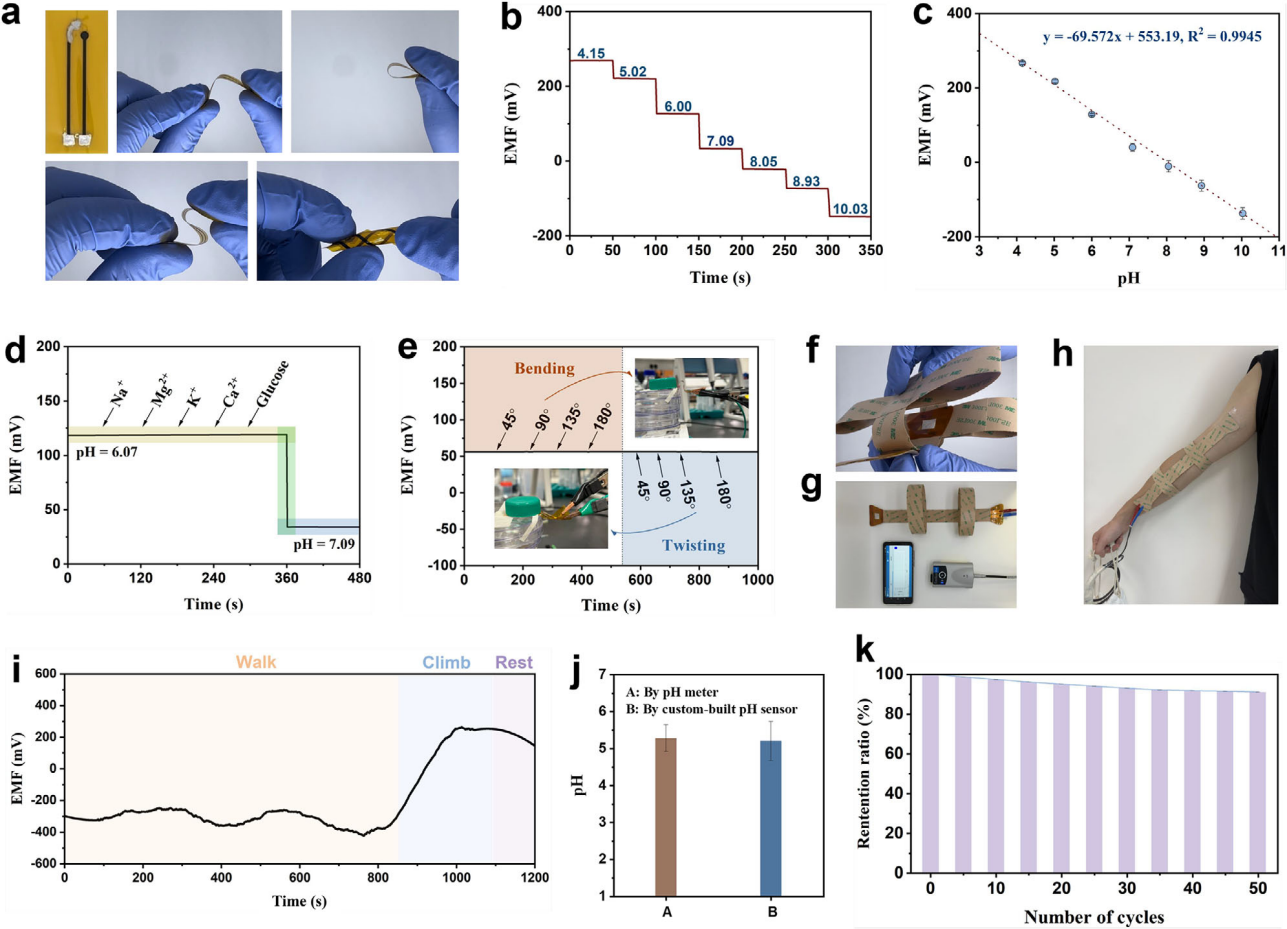
(a) Images of PI‐based electrode modified with P(ANI‐co‐MOANI) and its flexibility; (b) OCP versus pH curve and (c) calibration plot of P(ANI‐co‐MOANI)‐modified potentiometric pH sensor; (d) Interference and (e) bending, twisting studies of P(ANI‐co‐MOANI)‐modified potentiometric pH sensor; (f) The flexibility of integrated wearable transducer; (g) Main components for the pH monitoring through Bluetooth technology; (h) Real‐time pH monitoring through custom‐built wearable pH sensor; (i) OCP curve recorded during the continuous pH monitoring of sweat; (j) Comparison of pH value measured by pH meter and custom‐built pH sensor. The sweat samples are collected after exercise. Data are presented as mean ± SD. n = 3 from independent experiments. Error bars represent standard deviation; (k) Stability tests of custom‐built pH sensor.

The mechanical properties and practical pH sensing ability of advanced P(ANI‐co‐MOANI)‐based flexible and wearable pH sensor are evaluated. As seen from **Figure** **5**a, the potentiometric pH sensor exhibits excellent flexibility, capable of bending and twisting up to 180° without deformation. The pH sensitivity displays a wide dynamic pH range (4.15–10.03), achieving 69.572 mV pH^−1^ with a high linear relationship (R^2^ = 0.9945) (Figure 5b,c). The pH sensing performance challenged under the interference of various electrolytes and deformation conditions is further investigated (Figure 5d,e). The representative presence of Na^+^, Mg^2+^, K^+^, Ca^2+^ and glucose at their physiological concentrations in sweat has almost no impact on the OCP signal of the pH sensor.^[^ ^62^ ^]^ Moreover, different bending and twisting actions do not have an insignificant impact on the OCP signal. These results exhibit that the advanced P(ANI‐co‐MOANI)‐based flexible pH sensor has excellent anti‐interference performance.

After assessing the mechanical and pH sensing performance, the real‐time sweat pH monitoring using the custom‐built P(ANI‐co‐MOANI)‐based flexible and wearable pH sensor is performed. Participation is voluntary, and consent was obtained from the respondent prior to data collection. The measuring system includes a Bluetooth module, a smartphone, a self‐powered compact electrochemical workstation, and an integrated P(ANI‐co‐MOANI)‐based flexible and wearable pH sensor, making it portable for real‐time pH monitoring via a mobile application (Figure 5f–h). The corresponding results are shown in Figure 5i. During the initial walking stage, the OCP signal fluctuates between −200 and −400 mV due to the non‐sweating state. After walking for more than 800 s, the secretion of sweat on the skin leads to a rapid increase in the OCP signal detected by the wearable pH sensor. Subsequently, as the exercise intensity increases through climbing slopes, the measured OCP signal reaches a steady state, indicating a decrease in pH with reinforced exercise intensity, consistent with findings in sports medicine.^[^ ^28^ ^]^ During intense exercise, skeletal muscle cells produce a large amount of lactate through anaerobic respiration, leading to the pH decrease in body fluid. As climbing time continues to increase, the OCP signal gradually stabilizes. During the rest period after exercise, the OCP signal slowly decreases, reaching acid‐base balance due to the metabolism of lactate in the body. To measure the accuracy of the sensor, we test the pH of collected sweat using both a pH meter and the P(ANI‐co‐MOANI)‐based wearable pH sensor (Figure 5j). Experimental results depict excellent accuracy for the sensor (pH = 5.28 and 5.20, respectively). Furthermore, we evaluate the reusability of the integrated wearable transducer (Figure 5k). After 50 measurements, only 8.9% of the initial OCP signal decreases, indicating a good stability of the P(ANI‐co‐MOANI)‐based wearable sensor.

## 3 Conclusion

We have demonstrated both theoretically and experimentally the new concept on DFT‐assisted design and fabrication of high‐performance CPs with enhanced ionic molecular interactions. We have proven the concept via rational molecular design of aniline‐based donor‐acceptor CPs with enhanced molecular interactions between H^+^ and CPs. Various aniline‐based CPs were designed and assessed using DFT calculations. The typical D‐A P(ANI‐co‐MAONI), the D‐D P(ANI‐co‐FANI), and PANI exhibited different energy level distributions and molecular electrostatic potentials, revealing distinct electron transfer efficiencies. Furthermore, their pH sensing performances were experimentally verified, aligning well with the calculated results. The high‐performance D‐A type aniline‐based CP., i.e., the P(ANI‐co‐MAONI), significantly improves the sensitivity and stability of PANI‐based pH sensors by enhancing interactions with H^+^ ions, with improved pH sensitivity (65.193 mV pH^−1^), reproducibility (98.7%), and repeatability (93.2%), and excellent operational stability over 24 h compared to P(ANI‐co‐FANI) and PANI. Finally, we demonstrated the integration of the advanced D‐A type P(ANI‐co‐MAONI) into a flexible and durable transducer for real‐time sweat pH monitoring during various activities. The successful DFT‐assisted design and fabrication of high‐performance aniline‐based D‐A type CPs through rational molecular engineering provides a new concept and insight into designing advanced functional CPs, potentially extended to other types of PEDOT/PPy‐derived CPs with improved ionic molecular interactions, paving the way for the future development of advanced organic bioelectronic devices such as biosensors, wearable drug delivery, energy storage and biofuel cells.

## 4 Experimental Section

### Materials

Aniline, *o*‐methoxyaniline, *o*‐fluoroaniline, potassium chloride (KCl), sodium hydroxide (NaOH), polyvinyl alcohol (PVA), sodium chloride (NaCl), magnesium chloride (MgCl_2_), potassium chloride (KCl), calcium chloride (CaCl_2_), disodium hydrogen phosphate monohydrate (Na_2_HPO_4_∙H_2_O), potassium dihydrogen phosphate (KH_2_PO_4_), and glucose were purchased from Macklin (Shanghai, China). All reagents were analytical grade without further purification. The preparation of phosphate buffer solution (PBS 7.4) combined KH_2_PO_4_ and Na_2_HPO_4_. Conductive silver ink, silver/silver chloride (Ag/Ag/Cl) ink and polyimide film were purchased from Canrd. Ltd. (China) and Tecos Technology. Ltd. (China), respectively. Deionized (DI) water was obtained by the MilIi‐Q System.

### Fabrication of Aniline‐Based Engineered Conducting Polymers

The synthesis of various aniline‐based engineered conducting polymers was conducted using the electropolymerization method (Figure S8, Supporting Information) via an electrochemical workstation (Palmsens 4, with software PSTrace 5.9) within a three‐electrode system comprising a working electrode (i.e., glassy carbon electrode (GCE)), a reference electrode (Ag/AgCl), and a counter electrode (Pt wire). Before the electropolymerization, the working electrode (GCE) was polished using 0.3 and 0.05 µm aluminum powder and subsequently rinsed thoroughly with deionized water prior to use. P(ANI‐co‐MOANI) and P(ANI‐co‐FANI) were synthesized via chronoamperometry (10 min) at applied potentials of 0.74 and 0.95 V, respectively, in mixed solutions containing 0.1 M aniline and 0.1 M o‐methoxyaniline or o‐fluoroaniline, with 1 M HCl serving as the supporting electrolyte. Likewise, PANI was prepared using similar method, employing solely aniline (0.1 M), and at an applied potential of 0.80 V (10 min). Following electropolymerization, the deposited CPs on the working electrodes were subjected to extensive washing steps (five times with deionized water and five times with ethanol) to remove residual monomers. Afterward, the deposited CPs were air‐dried at room temperature and reserved for future applications.

### Integration of Custom‐Built Flexible pH Sensor in a Wearable Device

Laser‐Induced Graphene (LIG) was fabricated on a polyimide film by using a commercial CO_2_ laser cutting machine (Gweike Cloud RF, China) with different optimized parameters (Fixed power: 5 W; Scanning speed: 170 mm∙s^−1^; Defocus distance: 15 mm), the pattern of which could be found in Schedule 1. This LIG pattern was later functionalized by coating conductive silver and Ag/AgCl ink to serve as a two‐electrode system. After being cured at 60 °C for 30 min, 5 µL of PVB polymer containing KCl fine powder (30% w/v) was drop‐cast on the top of the reference electrode (RE) for another curation for 30 min at 60 °C. To further functionalize the fabricated two‐electrode system with pH sensing performance, the working electrode (WE) was then electrodeposited with P(ANI‐co‐MOANI) using the same method mentioned above.

### Physical Characterizations

The phase characteristics of various polymers were analyzed using X‐ray diffraction (XRD, Bruker Advance D8, Germany). Raman data (LabRAM HR Evolution, France) was obtained at a wavelength of 532 nm, ranging from 200 to 4000 cm^−1^. Elemental distribution and microstructure were recorded using scanning electron microscopy (SEM, JEOL‐6700F, Japan). The UV‐vis spectra of P(ANI‐co‐MOANI) at different pH levels were collected through the conducting polymers‐decorated thin film, ranging from 200 to 1000 nm, using a UV–vis spectrophotometer (Lambda 950, USA).

### Electrochemical Measurements

To evaluate the pH sensing performance of different polymers, open circuit potential (OCP) measurements between polymer‐modified GCE and RE were conducted at room temperature using an electrochemical workstation (PalmSens4, Netherlands) in PBS buffer solutions with varying pH levels. The pH value of the tested solution was confirmed using a commercial pH meter (FiveEasy Plus pH meter FP20, Switzerland).

### Molecular Simulation

Different polymer molecules with simplified unit were initially optimized using density functional theory (DFT) combined with the B3LYP/6‐31G* function on Gaussian. Their electrostatic potential (ESP), the lowest unoccupied molecular orbital (LUMO), the highest occupied molecular orbital (HOMO), and localized orbital locator (LOL) were further visualized for comprehensive structural analysis.^[^ ^63^, ^64^ ^]^

### On‐Body Sweat Sensing

After assembling the two‐electrode sensing module and integrating the sweat collection layer (cellulose membrane), this part was then encapsulated using flexible adhesive films. Conductive copper wires were used to connect the electrodes to a portable mobile sensing system. The entire device was then secured for stable on‐body operation. The on‐body test was conducted in three stages. Warm‐up walking: The participant began with ≈800 s light walk to initiate mild perspiration and ensure proper skin contact with the sensor. Incline climbing: The participant then performed stair climbing to induce active sweating. During this period, real‐time pH signal changes were continuously monitored via a mobile device interface connected to the sensing module. Rest period: After sufficient sweat generation, the participant stopped and rested. Signal stabilization and recovery were observed during this phase to evaluate the sensor's dynamic response and reversibility. The research experiments were approved by Shun Hing Institute of Advanced Engineering (BME‐p2‐23) at The Chinese University of Hong Kong. The informed consent form was obtained from the volunteer participant.

### Statistical Analysis

All quantitative data in this study were analyzed using Origin 2021. Electrochemical measurements were performed using a PalmSens4 workstation. For on‐body sweat pH sensing experiments, data were presented as mean ± standard deviation (SD), with a sample size of *n* = 3.

## Conflict of Interest

The authors declare no conflict of interest.

## Author Contributions

J.Q. wrote the original draft, curated the data, developed the methodology, and contributed to the conceptualization. R.S. conducted formal analysis. L.Z. conducted formal analysis. W.C.M. reviewed and edited the manuscript, contributed to conceptualization and supervision, and acquired funding.

## Supporting information



Supporting Information

## Data Availability

The data that support the findings of this study are available from the corresponding author upon reasonable request.
