# Supplementary material for: Rational Molecular Design of Aniline‐Based Donor‐Acceptor Conducting Polymers Enhancing Ionic Molecular Interaction for High‐Performance Wearable Bioelectronics
Source: Adv Healthc Mater. 2025 Jul 4;14(25):2501929. doi: 10.1002/adhm.202501929 (PMC12477569; doi:10.1002/adhm.202501929)
Supplement: Supplementary file 1 — Supporting Information [file ADHM-14-0-s001.pdf]

ADVANCED HEALTHCARE MATERIALS

Supporting Information

for *Adv. Healthcare Mater.*, DOI 10.1002/adhm.202501929

Rational Molecular Design of Aniline-Based Donor-Acceptor Conducting Polymers Enhancing Ionic Molecular Interaction for High-Performance Wearable Bioelectronics

*Junning Qian, Ruya Shi, Li Zhang and Wing Cheung Mak**

Supporting Information

Rational Molecular Design of Aniline-based Donor-Acceptor Conducting Polymers Enhancing Ionic Molecular Interaction for High-Performance Wearable Bioelectronics

Junning Qian[1,2], Ruya Shi[1], Li Zhang[1], Wing Cheung Mak[1,2,*]

1 Department of Biomedical Engineering, The Chinese University of Hong Kong, Shatin, New Territories, Hong Kong, China

2 Shun Hing Institute of Advanced Engineering, The Chinese University of Hong Kong, Shatin, New Territories, Hong Kong, China

[*]Corresponding author. E-mail: wing.cheung.mak@cuhk.edu.hk (Wing Cheung Mak)

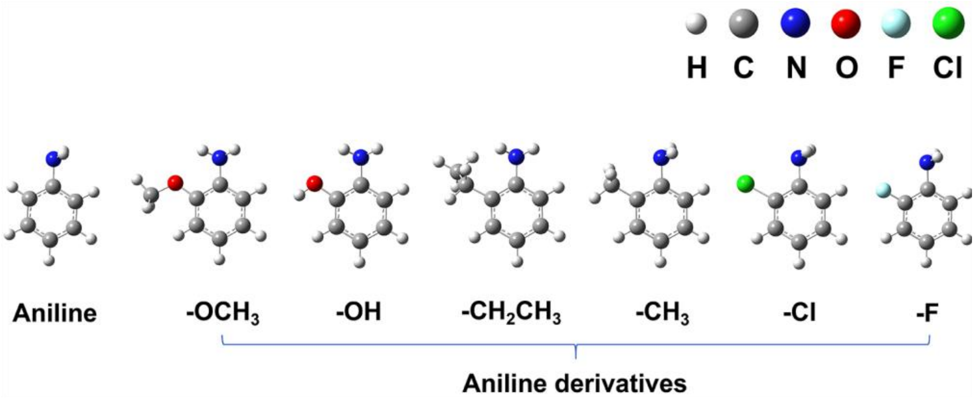

Figure S1. Molecular models of aniline and its derivatives.

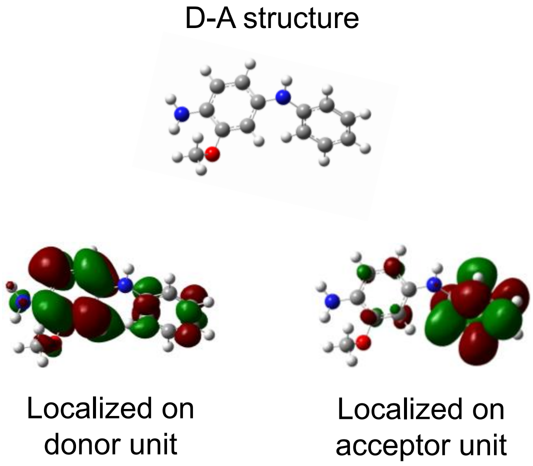

Figure S2. D-A structure and orbital energy levels localized on donor and acceptor units.

The orbital overlap integrals between these two orbitals were computed to be 0.385, indicating a significant degree of orbital overlap between the donor and acceptor units.

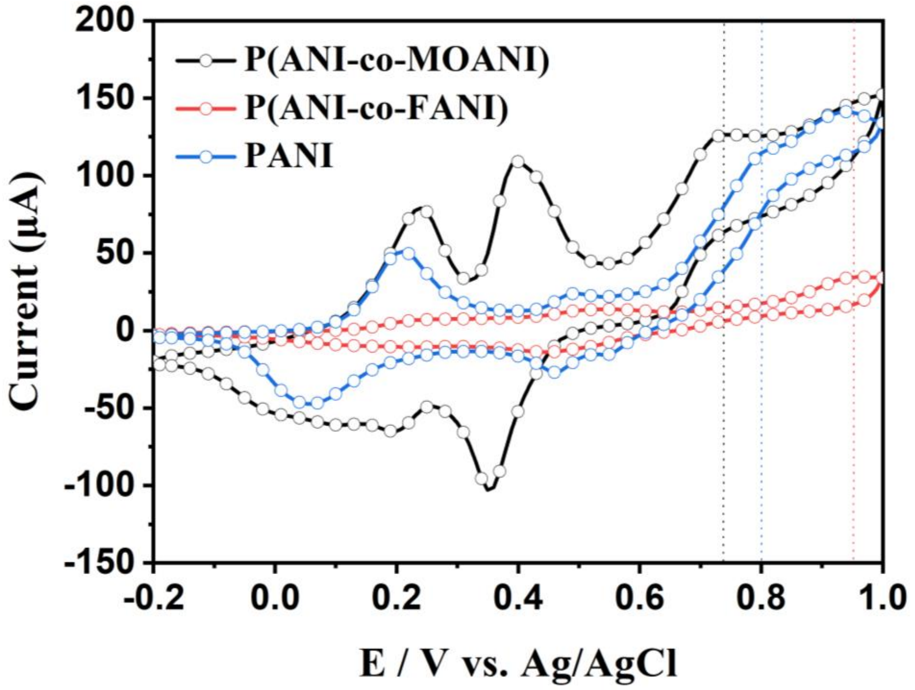

Figure S3. Cyclic voltammetry curves of P(ANI-co-MOANI), P(ANI-co-FANI) and PANI.

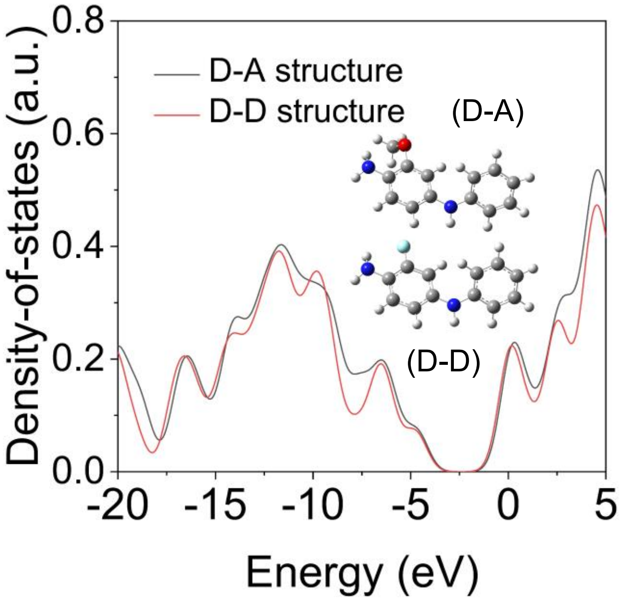

Figure S4. Density of states distributions of D-A and D-D polymers

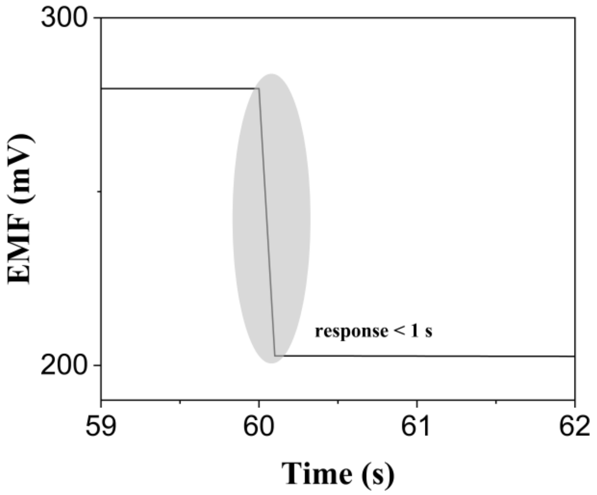

Figure S5. Response time of P(ANI-co-MOANI) in pH sensing.

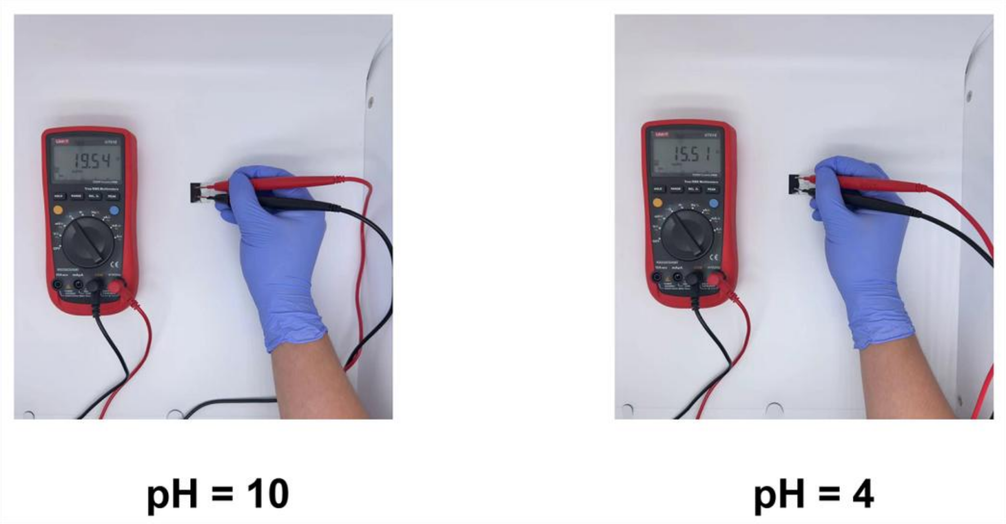

Figure S6. Resistances of P(ANI-co-MOANI)-modified FTO glass dealt with different solutions of pH 10 and 4.

Obviously, the resistances of P(ANI-co-MOANI)-modified FTO glass is decreased after it is dealt with the acid solution. This means the H^+-doping process increases the corresponding conductivity.

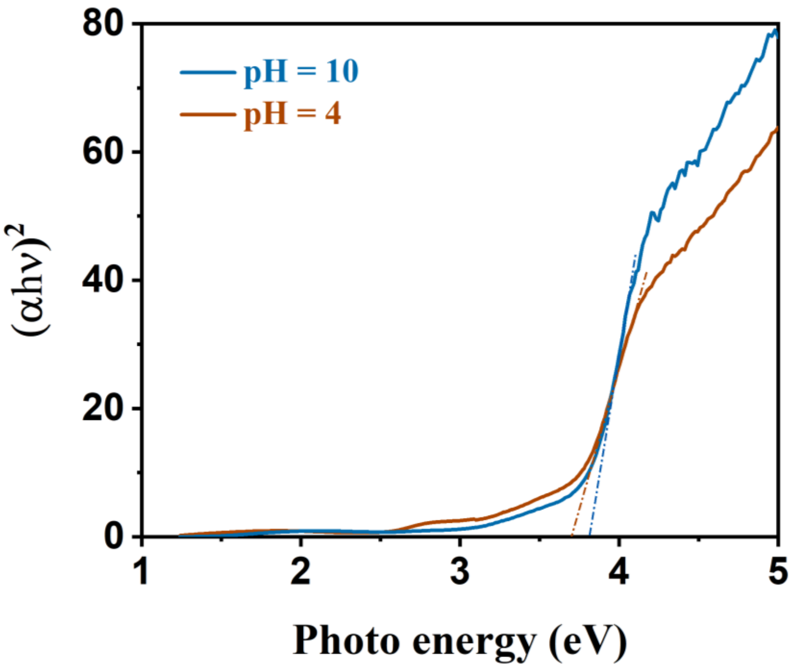

Figure S7. Tauc plots of P(ANI-co-MOANI)-modified ITO glass dealt with different solutions of pH 10 and 4.

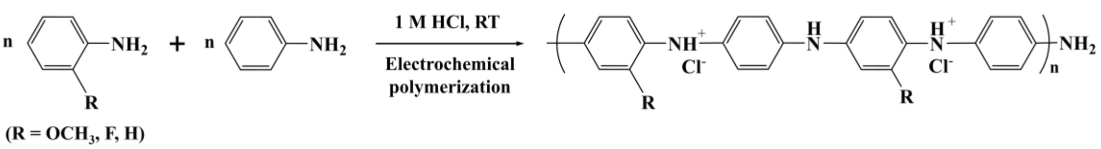

Figure S8. Electrochemical polymerization process of aniline-based copolymers.

Table S1. Sensing properties of recent potentiometric pH sensors reported in the literature

pH sensing materials	Electrolyte	Substrate/ Fabrication method	pH range and linearity	Sensitivity (mV/pH)	Response time (s)	Long-term stability	Mechanical durability (cycles)	Ref
SiO_2 coated carbon	0.1 M PBS	PET film/Vapor deposition	4-10	48.0	8	4 mV/24 h*	/	1
Graphite-polyurethane composite	0.1 M PBS	PDMS/Screen printing	5-10	13.76 ± 5.45	8	/	500	2
Polyaniline	0.1 M PBS	PU-PDMS/Electrodeposition	3-11	46.7	4.9	/	/	3
IrO_2	0.1 M PBS	Paper/Sputtering	4-7	61.0	3000	/	/	4
Polyaniline-Au-PMNA	0.1 M PBS	PEN film/Soft lithography	4-8.6	62.9	/	2.96 mV/24 h*	/	5
WO_3	0.1 M PBS	GCE/Coating	1-11	51.77	5	/	/	6
F-$Ti_3C_2T_x$/PANI	0.1 M PBS	PET film/ Screen-Printing	1-11	40.7	/	12.96 mV/24 h*	/	7
P(ANI-co-MOANI)	0.1 M PBS	GCE/Electrodeposition	4-10	**65.193**	< 1	3.36 mV/24 h	/	**This work**

* Remark: The open-circuit potential drift has been uniformly defined within 24 h.

References

1. Kasi, V.; Sedaghat, S.; Alcaraz, A. M.; Maruthamuthu, M. K.; Heredia-Rivera, U.; Nejati, S.; Nguyen, J.; Rahimi, R., Low-Cost Flexible Glass-Based pH Sensor via Cold Atmospheric Plasma Deposition. *ACS Appl Mater Interfaces* **2022,** *14* (7), 9697-9710.
2. Dang, W.; Manjakkal, L.; Navaraj, W. T.; Lorenzelli, L.; Vinciguerra, V.; Dahiya, R., Stretchable wireless system for sweat pH monitoring. *Biosens Bioelectron* **2018,** *107*, 192-202.
3. Wang, W.; Harimurti, S.; Inoue, D.; Nayeem, M. O. G.; Wang, J.; Okuda, C.; Hashizume, D.; Lee, S.; Fukuda, K.; Yokota, T.; Someya, T., Janus Membrane-Based Wearable pH Sensor with Sweat Absorption, Gas Permeability, and Self-Adhesiveness. *ACS Appl Mater Interfaces* **2024,** *16* (21), 27065-27074.
4. Nie, C.; Frijns, A.; Zevenbergen, M.; Toonder, J. d., An integrated flex-microfluidic-Si chip device towards sweat sensing applications. *Sensors and Actuators B: Chemical* **2016,** *227*, 427-437.
5. Dervisevic, M.; Dervisevic, E.; Esser, L.; Easton, C. D.; Cadarso, V. J.; Voelcker, N. H., Wearable microneedle array-based sensor for transdermal monitoring of pH levels in interstitial fluid. *Biosens Bioelectron* **2023,** *222*, 114955.
6. Tang, Y.; Gan, S.; Zhong, L.; Sun, Z.; Xu, L.; Liao, C.; Lin, K.; Cui, X.; He, D.; Ma, Y.; Wang, W.; Niu, L., Lattice Proton Intercalation to Regulate WO_3-Based Solid-Contact Wearable pH Sensor for Sweat Analysis. *Advanced Functional Materials* **2021,** *32* (4).
7. Chen, L.; Chen, F.; Liu, G.; Lin, H.; Bao, Y.; Han, D.; Wang, W.; Ma, Y.; Zhang, B.; Niu, L., Superhydrophobic Functionalized $Ti_3C_2T_x$ MXene-Based Skin-Attachable and Wearable Electrochemical pH Sensor for Real-Time Sweat Detection. *Anal Chem* **2022,** *94* (20), 7319-7328.
